# Research Project Evaluation—Learnings from the PATHWAYS Project Experience

**DOI:** 10.3390/ijerph15061071

**Published:** 2018-05-25

**Authors:** Aleksander Galas, Aleksandra Pilat, Matilde Leonardi, Beata Tobiasz-Adamczyk

**Affiliations:** 1 Epidemiology and Preventive Medicine, Jagiellonian University Medical College, 31-034 Krakow, Poland; aleksander.galas@uj.edu.pl (A.G.); aleksandra.pilat@uj.edu.pl (A.P.); 2 Fondazione IRCCS, Neurological Institute Carlo Besta, 20-133 Milano, Italy; matilde.Leonardi@istituto-besta.it

**Keywords:** public health, project process evaluation, internal evaluation, SWOT analysis, project achievements, project management and monitoring

## Abstract

Background: Every research project faces challenges regarding how to achieve its goals in a timely and effective manner. The purpose of this paper is to present a project evaluation methodology gathered during the implementation of the *Participation to Healthy Workplaces and Inclusive Strategies in the Work Sector* (the EU PATHWAYS Project). The PATHWAYS project involved multiple countries and multi-cultural aspects of re/integrating chronically ill patients into labor markets in different countries. This paper describes key project’s evaluation issues including: (1) purposes, (2) advisability, (3) tools, (4) implementation, and (5) possible benefits and presents the advantages of a continuous monitoring. Methods: Project evaluation tool to assess structure and resources, process, management and communication, achievements, and outcomes. The project used a mixed evaluation approach and included Strengths (S), Weaknesses (W), Opportunities (O), and Threats (SWOT) analysis. Results: A methodology for longitudinal EU projects’ evaluation is described. The evaluation process allowed to highlight strengths and weaknesses and highlighted good coordination and communication between project partners as well as some key issues such as: the need for a shared glossary covering areas investigated by the project, problematic issues related to the involvement of stakeholders from outside the project, and issues with timing. Numerical SWOT analysis showed improvement in project performance over time. The proportion of participating project partners in the evaluation varied from 100% to 83.3%. Conclusions: There is a need for the implementation of a structured evaluation process in multidisciplinary projects involving different stakeholders in diverse socio-environmental and political conditions. Based on the PATHWAYS experience, a clear monitoring methodology is suggested as essential in every multidisciplinary research projects.

## 1. Introduction

Over the last few decades, a strong discussion on the role of the evaluation process in research has developed, especially in interdisciplinary or multidimensional research [1,2,3,4,5]. Despite existing concepts and definitions, the importance of the role of evaluation is often underestimated. These dismissive attitudes towards the evaluation process, along with a lack of real knowledge in this area, demonstrate why we need research evaluation and how research evaluation can improve the quality of research. Having firm definitions of ‘evaluation’ can link the purpose of research, general questions associated with methodological issues, expected results, and the implementation of results to specific strategies or practices.

Attention paid to projects’ evaluation shows two concurrent lines of thought in this area. The first is strongly associated with total quality management practices and operational performance; the second focuses on the evaluation processes needed for public health research and interventions [6,7].

The design and implementation of process’ evaluations in fields different from public health have been described as multidimensional. According to Baranowski and Stables, process evaluation consists of eleven components: recruitment (potential participants for corresponding parts of the program); maintenance (keeping participants involved in the program and data collection); context (an aspect of environment of intervention); resources (the materials necessary to attain project goals); implementation (the extent to which the program is implemented as designed); reach (the extent to which contacts are received by the targeted group); barriers (problems encountered in reaching participants); exposure (the extent to which participants view or read material); initial use (the extent to which a participant conducts activities specified in the materials); continued use (the extent to which a participant continues to do any of the activities); contamination (the extent to which participants receive interventions from outside the program and the extent to which the control group receives the treatment) [8].

There are two main factors shaping the evaluation process. These are: (1) *what is evaluated* (whether the evaluation process revolves around project itself or the outcomes which are external to the project), and (2) *who is an evaluator* (whether an evaluator is internal or external to the project team and program). Although there are several existing gaps in current knowledge about the evaluation process of external outcomes, the use of a formal evaluation process of a research project itself is very rare.

To define a clear evaluation and monitoring methodology we performed different steps. The purpose of this article is to present experiences from the project evaluation process implemented in the *Participation to Healthy Workplaces and Inclusive Strategies in the Work Sector* (the EU PATHWAYS project. The manuscript describes key project evaluation issues as: (1) purposes, (2) advisability, (3) tools, (4) implementation, and (5) possible benefits. The PATHWAYS project can be understood as a specific case study—presented through a multidimensional approach—and based on the experience associated with general evaluation, we can develop patterns of good practices which can be used in other projects.

### 1.1. Theoretical Framework

The first step has been the clear definition of what is an *evaluation strategy or methodology*. The term *evaluation* is defined by the Cambridge Dictionary as the process of judging something’s quality, importance, or value, or a report that includes this information [9] or in a similar way by the Oxford Dictionary as the making of a judgment about the amount, number, or value of something [10]; assessment and in the activity, it is frequently understood as associated with the end rather than with the process. Stufflebeam, in its monograph, defines evaluation as a study designed and conducted to assist some audience to assess an object’s merit and worth. Considering this definition, there are four categories of evaluation approaches: (1) pseudo-evaluation; (2) questions and/or methods-oriented evaluation; (3) improvement/accountability evaluation; (4) social agenda/advocacy evaluation [11].

In brief, considering Stufflebeam’s classification, pseudo-evaluations promote invalid or incomplete findings. This happens when findings are selectively released or falsified. There are two pseudo-evaluation types proposed by Stufflebeam: (1) public relations-inspired studies (studies which do not seek truth but gather information to solicit positive impressions of program), and (2) politically controlled studies (studies which seek the truth but inappropriately control the release of findings to right-to-know audiences). 

The questions and/or methods-oriented approach uses rather narrow questions, which are oriented on operational objectives of the project. Questions oriented uses specific questions, which are of interest by accountability requirements or an expert’s opinions of what is important, while method oriented evaluations favor the technical qualities of program/process. The general concept of these two is that it is better to ask a few pointed questions well to get information on program merit and worth [11]. In this group, one may find the following evaluation types: (a) objectives-based studies: typically focus on whether the program objectives have been achieved through an internal perspective (by project executors); (b) accountability, particularly payment by results studies: stress the importance of obtaining an external, impartial perspective; (c) objective testing program: uses standardized, multiple-choice, norm-referenced tests; (d) outcome evaluation as value-added assessment: a recurrent evaluation linked with hierarchical gain score analysis; (e) performance testing: incorporates the assessment of performance (by written or spoken answers, or psychomotor presentations) and skills; (f) experimental studies: program evaluators perform a controlled experiment and contrast the outcomes observed; (g) management information system: provide information needed for managers to conduct their programs; (h) benefit-cost analysis approach: mainly sets of quantitative procedures to assess the full cost of a program and its returns; (i) clarification hearing: an evaluation of a trial in which role-playing evaluators competitively implement both a damning prosecution of a program—arguing that it failed, and a defense of the program—and arguing that it succeeded. Next, a judge hears arguments within the framework of a jury trial and controls the proceedings according to advance agreements on rules of evidence and trial procedures; (j) case study evaluation: focused, in-depth description, analysis, and synthesis of a particular program; (k) criticism and connoisseurship: certain experts in a given area do in-depth analysis and evaluation that could not be done in other way; (l) program theory-based evaluation: based on the theory beginning with another validated theory of how programs of a certain type within similar settings operate to produce outcomes (e.g., Health Believe Model, Predisposing, Reinforcing and Enabling Constructs in Educational Diagnosis and Evaluation and Policy, Regulatory, and Organizational Constructs in Educational and Environmental Development - thus so called PRECEDE-PROCEED model proposed by L. W. Green or Stage of Change Theory by Prochaska); (m) mixed method studies: include different qualitative and quantitative methods. 

The third group of methods considered in evaluation theory are improvement/accountability-oriented evaluation approaches. Among these, there are the following: (a) decision/accountability oriented studies: emphasizes that evaluation should be used proactively to help improve a program and retroactively to assess its merit and worth; (b) consumer-oriented studies: wherein the evaluator is a surrogate consumer who draws direct conclusions about the evaluated program; (c) accreditation/certification approach: an accreditation study to verify whether certification requirements have been/are fulfilled.

Finally, a social agenda/advocacy evaluation approach focuses on the assessment of difference, which is/was intended to be the effect of the program evaluation. The evaluation process in this type of approach works in a loop, starting with an independent evaluator who provides counsel and advice towards understanding, judging and improving programs as evaluations to serve the client’s needs. In this group, there are: (a) client-centered studies (or responsive evaluation): evaluators work with, and for, the support of diverse client groups; (b) constructivist evaluation: evaluators are authorized and expected to maneuver the evaluation to emancipate and empower involved and affected disenfranchised people; (c) deliberative democratic evaluation: evaluators work within an explicit democratic framework and uphold democratic principles in reaching defensible conclusions; (d) utilization-focused evaluation: explicitly geared to ensure that program evaluations make an impact.

### 1.2. Implementation of the Evaluation Process in the EU PATHWAYS Project

The idea to involve the evaluation process as an integrated goal of the PATHWAYS project was determined by several factors relating to the main goal of the project, defined as a special intervention to existing attitudes to occupational mobility and work activity reintegration of people of working age, suffering from specific chronic conditions into the labor market in 12 European Countries. Participating countries had different cultural and social backgrounds and different pervasive attitudes towards people suffering from chronic conditions.

The components of evaluation processes previously discussed proved helpful when planning the PATHWAYS evaluation, especially in relation to different aspects of environmental contexts. The PATHWAYS project focused on chronic conditions including: mental health issues, neurological diseases, metabolic disorders, musculoskeletal disorders, respiratory diseases, cardiovascular diseases, and persons with cancer. Within this group, the project found a hierarchy of patients and social and medical statuses defined by the nature of their health conditions. 

According to the project’s monitoring and evaluation plan, the evaluation process followed specific challenges defined by the project’s broad and specific goals and monitored the progress of implementing key components by assessing the effectiveness of consecutive steps and identifying conditions supporting the contextual effectiveness. Another significant aim of the evaluation component on the PATHWAYS project was to recognize the value and effectiveness of using a purposely developed methodology—consisting of a wide set of quantitative and qualitative methods. The triangulation of methods was very useful and provided the opportunity to develop a multidimensional approach to the project [12].

From the theoretical framework, special attention was paid to the explanation of medical, cultural, social and institutional barriers influencing the chance of employment of chronically ill persons in relation to the characteristics of the participating countries.

Levels of satisfaction with project participation, as well as with expected or achieved results and coping with challenges on local–community levels and macro-social levels, were another source of evaluation.

In the PATHWAYS project, the evaluation was implemented for an unusual purpose. This quasi-experimental design was developed to assess different aspects of the multidimensional project that used a variety of methods (systematic review of literature, content analysis of existing documents, acts, data and reports, surveys on different country-levels, deep interviews) in the different phases of the 3 years. The evaluation monitored each stage of the project and focused on process implementation, with the goal of improving every step of the project. The evaluation process allowed to perform critical assessments and deep analysis of benefits and shortages of the specific phase of the project. 

The purpose of the evaluation was to monitor the main steps of the Project, including the expectations associated with a multidimensional, methodological approach used by PATHWAYS partners, as well as improving communication between partners, from different professional and methodological backgrounds involved in the project in all its phases, so as to avoid errors in understanding the specific steps as well as the main goals. 

## 2. Materials and Methods 

The paper describes methodology and results gathered during the implementation of Work Package 3, Evaluation of the Participation to Healthy Workplaces and Inclusive Strategies in the Work Sector (the PATHWAYS) project. The work package was intended to keep internal control over the run of the project to achieve timely fulfillment of tasks, milestones, and purpose by all project partners.

### 2.1. Participants

The project consortium involved 12 partners from 10 different European countries. There were academics (representing cross-disciplinary research including socio-environmental determinants of health, clinicians), institutions actively working for the integration of people with chronic and mental health problems and disability, educational bodies (working in the area of disability and focusing on inclusive education), national health institutes (for rehabilitation of patients with functional and workplace impairments), an institution for inter-professional rehabilitation at a country level (coordinating medical, social, educational, pre-vocational and vocational rehabilitation), a company providing patient-centered services (in neurorehabilitation). All the partners represented vast knowledge and high-level expertise in the area of interest and all agreed with the World Health Organization’s (WHO) International Classification of Functioning, Disability and Health-ICF and of the biopsychosocial model of health and functioning. The consortium was created based on the following criteria:

- vision, mission, and activities in the area of project purposes,
- high level of experience in the area (supported by publications) and in doing research (being involved in international projects, collaboration with the coordinator and/or other partners in the past),
- being able to get broad geographical, cultural and socio-political representation from EU countries,
- represent different stakeholder type in the area.

### 2.2. Project Evaluation Tool

The tool development process involved the following steps:

(1) Review definitions of ‘evaluation’ and adopt one which consorts best with the reality of public health research area;
(2) Review evaluation approaches and decide on the content which should be applicable in the public health research;
(3) Create items to be used in the evaluation tool;
(4) Decide on implementation timing.

According to the PATHWAYS project protocol, an evaluation tool for the internal project evaluation was required to collect information about: (1) structure and resources; (2) process, management and communication; (3) achievements and/or outcomes and (4) SWOT analysis. A mixed methods approach was chosen. The specific evaluation process purpose and approach are presented in Table 1.

The tool was prepared following different steps. In the paragraph to assess structure and resources, there were questions about the number of partners, professional competences, assigned roles, human, financial and time resources, defined activities and tasks, and the communication plan. The second paragraph, process, management and communication, collected information about the coordination process, consensus level, quality of communication among coordinators, work package leaders, and partners, whether project was carried out according to the plan, involvement of target groups, usefulness of developed materials, and any difficulties in the project realization. Finally, the paragraph achievements and outcomes gathered information about project specific activities such as public-awareness raising, stakeholder participation and involvement, whether planned outcomes (e.g., milestones) were achieved, dissemination activities, and opinions on whether project outcomes met the needs of the target groups. Additionally, it was decided to implement SWOT analysis as a part of the evaluation process. SWOT analysis derives its name from the evaluation of Strengths (S), Weaknesses (W), Opportunities (O), and Threats (T) faced by a company, industry or, in this case, project consortium. SWOT analysis comes from the business world and was developed in the 1960s at Harvard Business School as a tool for improving management strategies among companies, institutions, or organization [13,14]. However, in recent years, SWOT analysis has been adapted in the context of research to improve programs or projects. 

For a better understanding of SWOT analysis, it is important to highlight the internal features of Strengths and Weaknesses, which are considered controllable. Strengths refers to work inside the project such as capabilities and competences of partners, whereas weaknesses refers to aspects, which needs improvement, such as resources. Conversely, Opportunities and Threats are considered outside factors and uncontrollable [15]. Opportunities are maximized to fit the organization’s values and resources and threats are the factors that the organization is not well equipped to deal with [9]. 

The PATHWAYS project members participated in SWOT analyses every three months. They answered four open questions about strengths, weaknesses, opportunities, and threats identified in evaluated period (last three months). They were then asked to assess those items on 10-point scale. The sample included results from nine evaluated periods from partners from ten different countries. 

The tool for the internal evaluation of the PATHWAYS project is presented in Appendix A.

### 2.3. Tool Implementation and Data Collection

The PATHWAYS on-going evaluation took place at three-month intervals. It consisted of on-line surveys, and every partner assigned a representative who was expected to have good knowledge on the progress of project’s progress. The structure and resources were assessed only twice, at the beginning (3rd month) and at the end (36th month) of the project. The process, management, and communication questions, as well as SWOT analysis questions, were asked every three months. The achievements and outcomes questions started after the first year of implementation (i.e., after 15th month), and some of items in this paragraph, (results achieved, whether project outcomes meet the needs of the target groups and published regular publications), were only implemented at the end of the project (36th month).

### 2.4. Evaluation Team

The evaluation team was created from professionals with different backgrounds and extensive experience in research methodology, sociology, social research methods and public health.

## 3. Results

The project started in 2015 and was carried out for 36 months. There were 12 partners in the PATHWAYS project, representing Austria, Belgium, Czech Republic, Germany, Greece, Italy, Norway, Poland, Slovenia and Spain and a European Organization. The on-line questionnaire was sent to all partners one week after the specified period ended and project partners had at least 2 weeks to fill in/answer the survey. Eleven rounds of the survey were performed.

The participation rate in the consecutive evaluation surveys was 11 (91.7%), 12 (100%), 12 (100%), 11 (91.7%), 10 (83.3%), 11 (91.7%), 11 (91.7%), 10 (83.3%), and 11 (91.7%) till the project end. Overall, it rarely covered the whole group, which may have resulted from a lack of coercive mechanisms at a project level to answer project evaluation questions.

### 3.1. Evaluation Results Considering Structure and Resources (3rd Month Only)

A total of 11 out of 12 project partners participated in the first evaluation survey. The structure and resources of the project were not assessed by the project coordinator and as such, the results in represent the opinions of the other 10 participating partners. The majority of respondents rated the project consortium as having at least adequate professional competencies. In total eight to nine project partners found human, financial and time resources ‘just right’ and the communication plan ‘clear’. More concerns were observed regarding the clarity of tasks, what is expected from each partner, and how specific project activities should be or were assigned.

### 3.2. Evaluation Results Considering Process, Management and Communication

The opinions about project coordination, communication processes (with coordinator, between WP leaders, and between individual partners/researchers) were assessed as ‘good’ and ‘very good’, along the whole period. There were some issues, however, when it came to the realization of specific goals, deliverables, or milestones of the project. 

**Figure 1:**
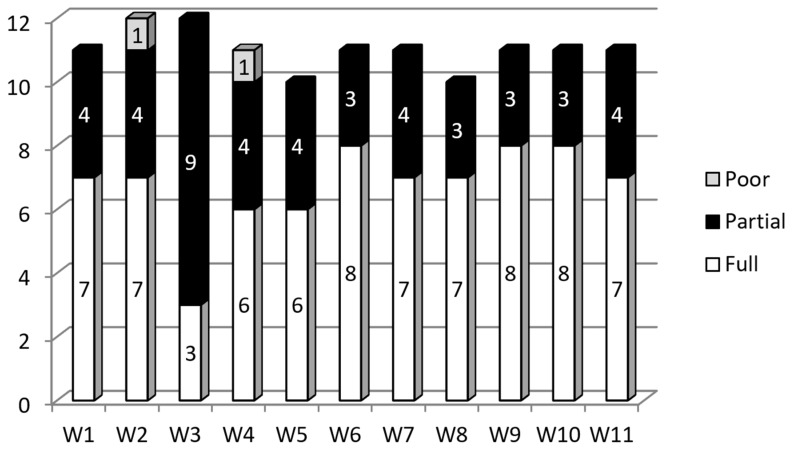
Partners’ opinions about the consensus around terms (shared glossary) in the project consortium across evaluation waves (W1—after 3-month realization period, and at 3-month intervals thereafter).

Given the broad scope of the project and participating partner countries, we created a glossary to unify the common terms used in the project. It was a challenge, as during the project implementation there were several discussions and inconsistencies in the concepts provided (Figure 1).

**Figure 2:**
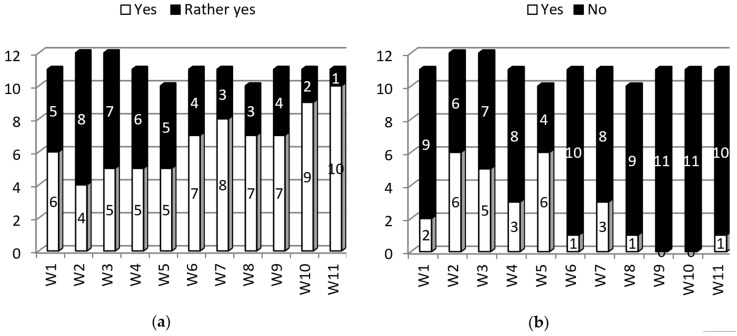
Partners’ reports on whether the project had been carried out according to the plan (**a**) and the experience of any problems in the process of project realization (**b**) (W1—after 3-month realization period, and at 3-month intervals thereafter).

**Figure 3:**
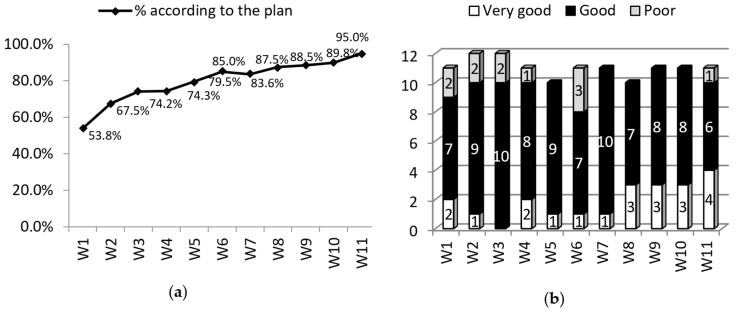
Partners’ reports on an approximate estimation (in percent) of the project plan implementation (what has been done according to the plan) (**a**) and the involvement of target groups (W1—after 3-month realization period, and at 3-month intervals thereafter) (**b**).

Other issues, which appeared during project implementation, were recruitment of, involvement with, and cooperation with stakeholders. There was a range of groups to be contacted and investigated during the project including individual patients suffering from chronic conditions, patients’ advocacy groups and national governmental organizations, policy makers, employers, and international organizations. It was found that during the project, the interest and the involvement level of the aforementioned groups was quite low and difficult to achieve, which led to some delays in project implementation (Figure 2). This was the main cause of smaller percentages of “what was expected to be done in designated periods of project realization time”. The issue was monitored and eliminated by intensification of activities in this area (Figure 3).

### 3.3. Evaluation Results Considering Achievements and Outcomes

The evaluation process was prepared to monitor project milestones and deliverables. One of the PATHWAYS project goals was to raise public awareness surrounding the reintegration of chronically ill people into the labor market. This was assessed subjectively by cooperating partners and only half (six) felt they achieved complete success on that measure. The evaluation process monitored planned outcomes according to: (1) determination of strategies for awareness rising activities, (2) assessment of employment-related needs, and (3) development of guidelines (which were planned by the project). The majority of partners completely fulfilled this task. Furthermore, the dissemination process was also carried out according to the plan.

### 3.4. Evaluation Results from SWOT

#### 3.4.1. Strengths

**Figure 4:**
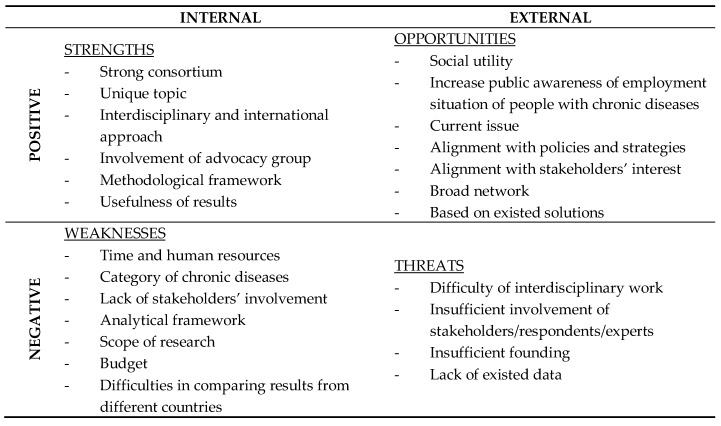
SWOT Analysis—a summary of main issues reported by PATHWAYS project partners.

Amongst the key issues identified across all nine evaluated periods (Figure 4), the “strong consortium” was highlighted as the most important strength of the PATHWAYS project. The most common arguments for this assessment were the coordinator’s experience in international projects, involvement of interdisciplinary experts who could guarantee a holistic approach to the subject, and a highly motivated team. This was followed by the uniqueness of the topic. Project implementers pointed to the relevance of the analyzed issues, which are consistent with social needs. They also highlighted that this topic concerned an unexplored area in employment policy. The interdisciplinary and international approach was also emphasized. According to the project implementers, the international approach allowed mapping of vocational and prevocational processes among patients with chronic conditions and disability throughout Europe. The interdisciplinary approach, on the other hand, enabled researchers to create a holistic framework that stimulates innovation by thinking across boundaries of particular disciplines—especially as the PATHWAYS project brings together health scientists from diverse fields (physicians, psychologists, medical sociologists, etc.) from ten European countries. This interdisciplinary approach is also supported by the methodology, which is based on a mixed-method approach (qualitative and quantitative data). The involvement of an advocacy group was another strength identified by the project implementers. It was stressed that the involvement of different types of stakeholders increased validity and social triangulation. It was also assumed that it would allow for the integration of relevant stakeholders. The last strength, the usefulness of results, was identified only in the last two evaluation waves, when the first results had been measured.

#### 3.4.2. Weaknesses

The survey respondents agreed that the main weaknesses of the project were time and human resources. The subject of the PATHWAYS project turned out to be very broad, and therefore the implementers pointed to the insufficient human resources and inadequate time for the implementation of individual tasks, as well as the project overall. This was related to the broad categories of chronic diseases chosen for analysis in the project. On one hand, the implementers complained about the insufficient number of chronic diseases taken into account in the project. On the other hand, they admitted that it was not possible to cover all chronic diseases in details. The scope of the project was reported as another weakness. In the successive waves of evaluation, the implementers more often pointed out that it was hard to cover all relevant topics. 

Nevertheless, some of the major weaknesses reported during the project evaluation were methodological problems. Respondents pointed to problems with the implementation of tasks on a regular basis. For example, survey respondents highlighted the need for more open questions in the survey that the questionnaire was too long or too complicated, that the tools were not adjusted for relevancy in the national context, etc. Another issue was that the working language was English, but all tools or survey questionnaire needed to be translated into different languages and this issue was not always considered by the Commission in terms of timing and resources. This issue could provide useful for further projects, as well as for future collaborations.

The difficulties of involving stakeholders were reported, especially during tasks, which required their active commitment, like participation in in-depth interviews or online questionnaires. Interestingly, the international approach was considered both strength and weakness of the project. The implementers highlighted the complexity of making comparisons between health care and/or social care in different countries. The budget was also identified as a weakness by the project implementers. More funds obtained from the partners could have helped PATHWAYS enhance dissemination and stakeholders’ participation.

#### 3.4.3. Opportunities

A list of seven issues within the opportunities category reflects the positive outlook of survey respondents from the beginning of the project to its final stage. Social utility was ranked as the top opportunity. The implementers emphasized that the project could fill a gap between the existing solutions and the real needs of people with chronic diseases and mental disorders. The implementers also highlighted the role of future recommendations, which would consist of proposed solutions for professionals, employees, employers, and politicians. These advantages are strongly associated with increasing awareness of employment situations of people with chronic diseases in Europe and the relevance of the problem. Alignment with policies, strategies, and stakeholders’ interests were also identified as opportunities. The topic is actively discussed on the European and national level, and labor market and employment issues are increasingly emphasized in the public discourse. What is more relevant is that the European Commission considers the issue crucial, and the results of the project are in line with its requests for the future. The implementers also observed increasing interest from the stakeholders, which is very important for the future of the project. Without doubt, the social network of project implementers provides a huge opportunity for the sustainability of results and the implementation of recommendations.

#### 3.4.4. Threats

Insufficient response from stakeholders was the top perceived threat selected by survey respondents. The implementers indicated that insufficient involvement of stakeholders resulted in low response rates in the research phase, which posed a huge threat for the project. The interdisciplinary nature of the PATHWAYS project was highlighted as a potential threat due to differences in technical terminology and different systems of regulating the employment of persons with reduced work capacity in each country, as well as many differences in the legislation process. Insufficient funding and lack of existing data were identified as the last two threats.

**Figure 5:**
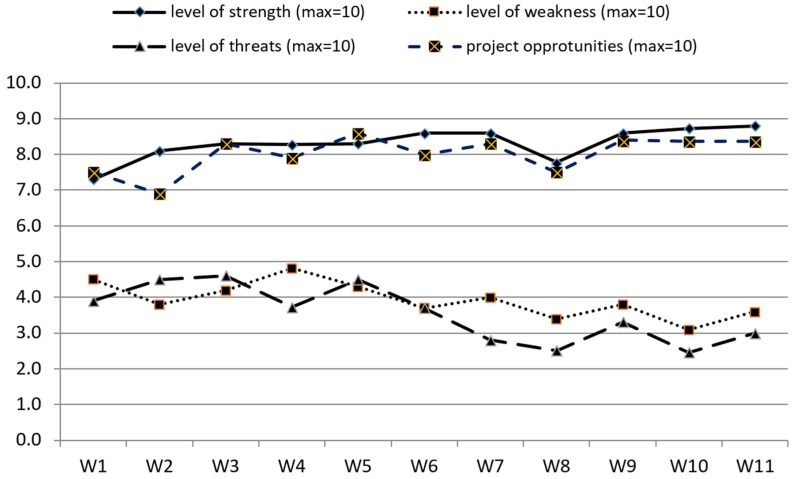
Numerical SWOT, combined, over a period of 36 months of project realization (W1—after 3-month realization period, and at 3-month intervals thereafter).

One novel aspect of the evaluation process in the PATHWAYS project was a numerical SWOT analysis. Participants were asked to score strengths, weaknesses, opportunities, and threats from 0 (meaning the lack of/no strengths, weaknesses) to 10 (meaning a lot of ... several ... strengths, weaknesses). This concept enabled us to get a subjective score of how partners perceive the PATHWAYS project itself and the performance of the project, as well as how that perception changes over time. Data showed an increase in both strengths and opportunities and a decrease in weaknesses and threats over the course of project implementation (Figure 5).

## 4. Discussion

The need for project evaluation was born from an industry facing challenges regarding how to achieve market goals in more efficient way. Nowadays, every process, including research project implementation, faces questions regarding its effectiveness and efficiency. 

The challenge of a research project evaluation is that the majority of research projects are described as unique, although we believe several projects face similar issues and challenges as those observed in the PATHWAYS project.

The main objectives of the PATHWAYS Project were (a) to identify integration and re-integration strategies that are available in Europe and beyond for individuals with chronic diseases and mental disorders experiencing work-related problems (such as unemployment, absenteeism, reduced productivity, stigmatization), (b) to determine their effectiveness, (c) to assess the specific employment-related needs of those people, and (d) to develop guidelines supporting the implementation of effective strategies of professional integration and reintegration. The broad area of investigation, partial knowledge in the field, diversity of determinants across European Union countries, and involvement with stakeholders representing different groups caused several challenges in the project, including:

- *problem*: uncovered, challenging, demanding (how to encourage stakeholders to participate, share experiences),
- *diversity*: different European regions; different determinants: political, social, cultural; different public health and welfare systems; differences in law regulations; different employment policies and issues in the system,
- *multidimensionality* of research: some quantitative, qualitative studies including focus groups, opinions from professionals, small surveys in target groups (workers with chronic conditions).

The challenges to the project consequently led to several key issues, which should be taken, into account during project realization:

- *partners*: with their own expertise and interests; different expectations; different views on what is more important to focused on and highlighted;
- *issues associated with unification*: between different countries with different systems (law, work-related and welfare definitions, disability classification, others);
- *coordination*: as multidimensionality of the project may have caused some research activities by partners to move in a wrong direction (data, knowledge which is not needed for the project purposes), a lack of project vision in (some) partners might postpone activities through misunderstanding;
- *exchange of information*: multidimensionality, the fact that different tasks were accomplished by different centers and obstacles to data collection required good communication methods and smooth exchange of information.

### Identified Issues and Implemented Solutions

There were several issues identified through the semi-internal evaluation process performed during the project. Those, which might be more relevant for the project realization, are mentioned in the Table 2. 

The PATHWAYS project included diverse partners representing different areas of expertise and activity (considering broad aspect of chronic diseases, decline in functioning and of disability, and its role in a labor market) in different countries and social security systems, which caused a challenge when developing a common language to achieve effective communication and better understanding of facts and circumstances in different countries. The implementation of continuous project process monitoring, and proper adjustment, enabled the team to overcome these challenges.

The evaluation tool has several benefits. First, it covers all key areas of the research project including structure and available resources, the run of the process, quality and timing of management and communication, as well as project achievements and outcomes. Continuous evaluation of all of these areas provides in-depth knowledge about project performance. Second, the implementation of SWOT tool provided opportunities to share out good and bad experiences by all project partners, and the use of a numerical version of SWOT provided a good picture about inter-relations strengths—weaknesses and opportunities—threats in the project and showed the changes in their intensity over time. Additionally, numerical SWOT may verify whether perception of a project improves over time (as was observed in the PATHWAYS project) showing an increase in strengths and opportunities and a decrease in weaknesses and threats. Third, the intervals in which partners were ‘screened’ by the evaluation questionnaire seems to be appropriate, as it was not very demanding but frequent enough to diagnose on-time some issues in the project process.

The experiences with the evaluation also revealed some limitations. There were no coercive mechanisms for participation in the evaluation questionnaires, which may have caused a less than 100% response rate in some screening surveys. Practically, that was not a problem in the PATHWAYS project. Theoretically, however, this might lead to unrevealed problems, as partners experiencing troubles might not report them. Another point is asking about quality of the consortium to the project coordinator, which has no great value (the consortium is created by the coordinator in the best achievable way and it is hard to expect other comments especially at the beginning of the project). Regarding the tool itself, the question Could you give us approximate estimation (in percent) of the project plan realization (what has been done according to the plan)? was expected to collect information about the project partners collecting data on what has been done out of what should be done during each evaluation period, meaning that 100% was what should be done in 3-month time in our project. This question, however, was slightly confusing at the beginning, as it was interpreted as percentage of all tasks and activities planned for the whole duration of the project. Additionally, this question only works provided that precise, clear plans on the type and timing of tasks were allocated to the project partners. Lastly, there were some questions with very low variability in answer types across evaluation surveys (mainly about coordination and communication). Our opinion is that if the project runs/performs in a smooth manner, one may think such questions useless, but in more complicated projects, these questions may reveal potential causes of troubles.

## 5. Conclusions

The PATHWAYS project experience shows a need for the implementation of structured evaluation processes in multidisciplinary projects involving different stakeholders in diverse socio-environmental and political conditions. Based on the PATHWAYS experience, a clear monitoring methodology is suggested as essential in every project and we suggest the following steps while doing multidisciplinary research:

1. Define area/s of interest (decision maker level/s; providers; beneficiaries: direct, indirect),
2. Identify 2–3 possible partners for each area (chain sampling easier, more knowledge about; check for publications),
3. Prepare a research plan (propose, ask for supportive information, clarify, negotiate),
4. Create a cross-partner groups of experts,
5. Prepare a communication strategy (communication channels, responsible individuals, timing),
6. Prepare a glossary covering all the important issues covered by the research project,
7. Monitor the project process and timing, identify concerns, troubles, causes of delays,
8. Prepare for the next steps in advance, inform project partners about the upcoming activities,
9. Summarize, show good practices, successful strategies (during project realization, to achieve better project performance).

## Figures and Tables

**Table 1:** Evaluation purposes and approaches adopted for the purpose in the PATHWAYS project.

Purpose of the Evaluation in the PATHWAYS Project	Approach Adopted	No If Items (Questions) Created *
*structure and resources* - number of partners - professional competencies - roles defined - human, financial, time resources	question oriented and management information system	6
*project activities* - plan - tasks - activities required	question oriented and management information system	3
*process, management, communication (timing, quality)* - communication with coordinator - communication with/between WP leaders - communication with/between project partners - consensus between partners - difficulties experienced	question oriented and management information system and improvement/accountability oriented	10
*project outcomes* - project carried according to the plan - target groups involvement - usefulness of developed materials	objective-based and outcome evaluation as value-added assessment and client-centered	3
*project achievements* - educational and public-awareness raising activities - milestones/deliverables achieved - stakeholders’ participation achieved - dissemination process - project results	objective based and case-study and accountability oriented	10

* Open ended questions are not counted here.

**Table 2:** Issues identified by the evaluation process and solutions implemented.

Issue	Comment	Solution/s
Clarity of tasks, what is expected from each partner, and how specific project activities are assigned	Each partner had a final copy of the PATHWAYS project proposal with a description of activities in each WP. Next specific tasks planned in each WP were presented, discussed and explained during the kick-off meeting	* Discuss the issue during the nearest project teleconference. WP leaders were obliged to explain/provide more details on what was planned to be prepared by each contributing partner * Provide detailed descriptions of each task and explanations what is expected as a result, and what type of the information is intended to be collected and analyzed * Clarify on what contribution is needed/expected from each partner
* timing * stakeholder involvement	Project tasks and WP coordinators agreed before the submission of the project for funding. Timetable was intensively discussed and agreed. The timing, deliverables and milestones were put into the Gantt Chart. All issues were discussed and clarified during the kick-off meeting	The main doubts about project resources and timing appeared during the realization of the project and were mainly caused by low levels of stakeholder participation and involvement. Successful strategies were presented by other participants Coordinators were expected to be monitored carefully during the project realization
Glossary	There was not a specific/named task to prepare a common glossary during project implementation. It came as a consequence of variability in terms of definitions regarding disability, labor sector, low regulations, and worker rights across participating European countries	* Consider and analyze variability in the area of research (especially if multidimensional and/or multicultural and/or cross country) * Recognize experts, prepare a team responsible for unification/standardization of issues considered (definitions, determinants, outcomes, processes)
Broad area of research (broad purposes, several diseases) meaning some partners had no expertise in every disease and reintegration strategies	The research team was created to get representatives of different expert groups in the investigated area	* Prepare a knowledge exchange plan across project partners * Share experience and concepts between partners * Monitor, ask about difficulties * Save some time for unexpected delays

## Appendix A

**Table A1:** The evaluation questionnaire developed for the PATHWAYS Project.

1. Structure and Resources (Questions to Be Asked at the Beginning of the Project and 36th Month)	2. Process, Management and Communication (Questions to Be Asked Every 3 Months; with Exception of Question 2.10)	3. Achievements/Outcomes (Questions to Be Asked Every 3 Months after the First Year; with Exception of Questions 3.5, 3.6, and 3.7)
1.1. The number of partners (institutions) in the consortium is: adequate/too many/too few Comments/potential improvements	2.1. The coordination of the project in the past 3 months has been: Very good/good/poor/very poor Comments/potential improvements	3.1. The educational and public-awareness raising activities that have been carried out in my country were successful: Yes, completely/only partly/not at all Comments/potential improvements List educational and public awareness-raising activities based on the project that have been undertaken (i.e., publications in press/in the internet, workshops, professional conferences, etc.)
1.2. In your opinion professional competences of the members of the consortium are: adequate/satisfactory enough/not enough Comments/potential improvements	2.2. The consensus around terms (shared glossary) in the project consortium seems to be: Full/partial/poor Comments/potential improvements	3.2. Have been the planned outcomes (milestones and deliverables) achieved (so far) according to: (a) identification of strategies Yes, completely/only partly/not at all Comments/potential improvements (b) determination of effectiveness of these strategies Yes, completely/only partly/not at all Comments/potential improvements (c) assessment of employment-related needs Yes, completely/only partly/not at all Comments/potential improvements (d) development of guidelines Yes, completely/only partly/not at all Comments/potential improvements
1.3. The roles of participants were defined: Very clear/relatively clear/unclear Comments/potential improvements	2.3. The process of implementation of research phases was: Very good/good/poor/very poor Comments/potential improvements	3.3. Have been relevant stakeholders participation achieved (so far)? All of them/most of them/some of them/none of them At the moment, how many contacts were successfully established against the planed (in percent) Comments/potential improvements
1.4. The resources foreseen for this project were: (a) human resources: too much/just right/not sufficient Comments/potential improvements (b) financial resources: too much/just right/not sufficient Comments/potential improvements (c) time resources: too much/just right/not sufficient Comments/potential improvements	2.4. How would you rate the communication with the coordinator of the project? (a) in terms of timing? Very good/good/poor/very poor (b) in terms of quality of information? Very good/good/poor/very poor Comments/potential improvements	3.4. Is the dissemination process carried out according to the plan? Completely/only partly/not at all Comments/potential improvements
1.5. Is the plan of the communication in the project clear? yes/no Comments/potential improvements	2.5. How would you rate the communication with WP leaders? (a) in terms of timing? Very good/good/poor/very poor (b) in terms of quality of information? Very good/good/poor/very poor Comments/potential improvements	3.5. The results of PATHWAYS project have been achieved (questions to be asked in 36th month): Completely/only partly/not at all Comments/potential improvement (questions to be asked in 36th month)
1.6. The tasks in research phases were defined: (a) in terms of clarity of what is expected to be done Very well/well/poor/very poor Comments/potential improvements (b) in terms of division of work Very well/well/poor/very poor Comments/potential improvements	2.6. How would you rate the communication between partners? (a) in terms of timing? Very good/good/poor/very poor (b) in terms of quality of information? Very good/good/poor/very poor Comments/potential improvements	3.6. Do project outcomes meet the needs of the target groups? Yes, completely/only partly/not at all Comments/potential improvements (questions to be asked in 36th month)
1.7. The activities to be carried out by each partner were: (a) defined: Very good/good/poor/very poor (b) implemented Very good/good/poor/very poor Comments/potential improvements	2.7. Has been the project carried out according to the plan? Yes/rather yes/rather no/no Could you give us approximate estimation (in percent) of the project plan realization (what has been done according to the plan) Comments/potential improvements	3.7. Please, list scientific publications and conference presentations based on the project that have been published/delivered (only those, where member of your team is the first author/presenter) (questions to be asked in 36th month)
	2.8. How would you rate the involvement of target groups Very good/good/poor/very poor Comments/potential improvements	
	2.9. How would you rate the usefulness of the developed materials (leaflets, information on the website etc)? Very good/good/poor/very poor Comments/potential improvements	
	2.10. The organization of project meetings was carried out: Very good/good/poor/very poor Comments/potential improvements (questions to be asked after team meetings)	
	2.11. Have you experienced any difficulties in the process of project realization? Yes/no If yes, describe briefly	

SWOT analysis:

What are strengths and weaknesses of the project? (list, please)

What are threats and opportunities? (list, please)

Visual SWOT:

Please, rate the project on the following continua:

How would you rate:

* strengths of the project?
  (no strengths) 0 1 2 3 4 5 6 7 8 9 10 (a lot of strengths, very strong)
* weaknesses of the project?
  (no weaknesses) 0 1 2 3 4 5 6 7 8 9 10 (a lot of weaknesses, very weak)
* risk of threats to the project?
  (no risks) 0 1 2 3 4 5 6 7 8 9 10 (several risks, inability to accomplish the task(s))
* project opportunities
  (no opportunities) 0 1 2 3 4 5 6 7 8 9 10 (project has a lot of opportunities)
